# Interleukin-1β mediates alterations in mitochondrial fusion/fission proteins and memory impairment induced by amyloid-β oligomers

**DOI:** 10.1186/s12974-021-02099-x

**Published:** 2021-02-21

**Authors:** Andre F. Batista, Tayná Rody, Leticia Forny-Germano, Suzana Cerdeiro, Maria Bellio, Sergio T. Ferreira, Douglas P. Munoz, Fernanda G. De Felice

**Affiliations:** 1 Institute of Medical Biochemistry Leopoldo de Meis, Federal University of Rio de Janeiro, CCS, room H2-019, Rio de Janeiro, RJ 21941-590 Brazil; 2 Department of Immunology, Institute of Microbiology Paulo de Góes, Federal University of Rio de Janeiro, Rio de Janeiro, RJ 21941-902 Brazil; 3 Institute of Biophysics Carlos Chagas Filho, Federal University of Rio de Janeiro, Rio de Janeiro, RJ 21941-902 Brazil; 4 Centre for Neuroscience Studies, Queen’s University, Kingston, Ontario K7L3N6 Canada; 5 Department of Psychiatry, Queen’s University, Kingston, Ontario K7L3N6 Canada

**Keywords:** Alzheimer’s disease, Mitochondrial dynamics, Mitochondrial dysfunction, IL-1β, Neuroinflammation, Aβ oligomers

## Abstract

**Background:**

The lack of effective treatments for Alzheimer’s disease (AD) reflects an incomplete understanding of disease mechanisms. Alterations in proteins involved in mitochondrial dynamics, an essential process for mitochondrial integrity and function, have been reported in AD brains. Impaired mitochondrial dynamics causes mitochondrial dysfunction and has been associated with cognitive impairment in AD. Here, we investigated a possible link between pro-inflammatory interleukin-1 (IL-1), mitochondrial dysfunction, and cognitive impairment in AD models.

**Methods:**

We exposed primary hippocampal cell cultures to amyloid-β oligomers (AβOs) and carried out AβO infusions into the lateral cerebral ventricle of cynomolgus macaques to assess the impact of AβOs on proteins that regulate mitochondrial dynamics. Where indicated, primary cultures were pre-treated with mitochondrial division inhibitor 1 (mdivi-1), or with anakinra, a recombinant interleukin-1 receptor (IL-1R) antagonist used in the treatment of rheumatoid arthritis. Cognitive impairment was investigated in C57BL/6 mice that received an intracerebroventricular (i.c.v.) infusion of AβOs in the presence or absence of mdivi-1. To assess the role of interleukin-1 beta (IL-1β) in AβO-induced alterations in mitochondrial proteins and memory impairment, interleukin receptor-1 knockout (*Il1r1*^*−/−*^) mice received an i.c.v. infusion of AβOs.

**Results:**

We report that anakinra prevented AβO-induced alteration in mitochondrial dynamics proteins in primary hippocampal cultures. Altered levels of proteins involved in mitochondrial fusion and fission were observed in the brains of cynomolgus macaques that received i.c.v. infusions of AβOs. The mitochondrial fission inhibitor, mdivi-1, alleviated synapse loss and cognitive impairment induced by AβOs in mice. In addition, AβOs failed to cause alterations in expression of mitochondrial dynamics proteins or memory impairment in *Il1r1*^*−/−*^ mice.

**Conclusion:**

These findings indicate that IL-1β mediates the impact of AβOs on proteins involved in mitochondrial dynamics and that strategies aimed to prevent pathological alterations in those proteins may counteract synapse loss and cognitive impairment in AD.

**Supplementary Information:**

The online version contains supplementary material available at 10.1186/s12974-021-02099-x.

## Introduction

Alzheimer’s disease (AD) is characterized by progressive memory loss and brain dysfunction that results from deterioration of synapses and brain cells [1, 2]. Accumulation of extracellular amyloid plaques, intraneuronal neurofibrillary tangles, and oxidative stress are among the most prominent alterations observed in AD brains [2]. Oxidative stress occurs when mitochondrial function is impaired [3–5]. Mitochondrial function requires proper mitochondrial dynamics, which comprises continuous and balanced processes of mitochondrial fission, fusion, and transport [6]. In mammalian cells, mitochondrial fusion is controlled by two outer-membrane-localized proteins, mitofusins 1 and 2 (MFN-1 and MFN-2), and one inner-membrane-localized protein, optic atrophy 1 (OPA-1) [6]. Mitochondrial fission, on the other hand, is mainly controlled by dynamin-related protein 1 (Drp1), which is predominantly localized in the cytosol and is recruited to the mitochondrial outer membrane to drive this process [6].

Several studies have indicated that mitochondria are important for synaptic development and plasticity [7–10], with mitochondria fission required for the establishment and maintenance of long-term potentiation [8]. Impaired mitochondrial dynamics has been implicated in neurodegenerative disorders including Alzheimer’s, Parkinson’s, and Huntington’s diseases [10–17]. Reduced expression of OPA-1, MFN-1, and MFN-2 and increased expression of fission protein 1 (Fis-1) and Drp1 have been reported in post-mortem AD brains [12, 18]. Mitochondrial dysfunction has further been observed in mouse models of AD [10, 12, 13, 19]. An elegant recent study showed that Drp1 inhibition protects against mitochondrial dysfunction and cognitive impairment in a mouse model of AD [13]. Amyloid-β oligomers (AβOs), toxins that accumulate in the brains of AD patients and impair synaptic function and structure [2, 20], have been shown to induce mitochondrial fragmentation and dysfunction in neurons [12, 13, 21].

Brain inflammation is an important feature of AD [22–24]. Specifically, the interleukin-1 beta (IL-1β) pathway has been implicated in the pathogenesis of AD [25–29]. Studies have shown elevated IL-1β in the brain, cerebrospinal fluid, and plasma of AD patients [30–32]. Interestingly, IL-1β and its receptor, interleukin-1 receptor (IL-1R), are predominantly expressed in the hippocampus, consistent with a major role of IL-1β signaling in modulating hippocampal memory function [33].

The mechanisms underlying altered neuronal mitochondrial dynamics in AD remain poorly understood. Because AβOs have been shown to trigger an increase in brain IL-1β levels [25, 34], we hypothesized that abnormal activation of this pro-inflammatory pathway could lead to synapse loss and memory impairment via alterations in mitochondrial dynamics. We show that anakinra, an IL-1R antagonist used in the treatment of rheumatoid arthritis [35, 36], protected against alterations in proteins involved in mitochondrial fission and fusion induced by AβOs in hippocampal cultures. Alterations in mitochondrial proteins were observed in the frontal cortex of cynomolgus macaques that received intracerebroventricular (i.c.v.) infusions of AβOs. Additionally, we found that mdivi-1, an inhibitor of mitochondrial fission that further affects cellular physiology in ways that are independent of mitochondrial fusion [37–41], prevented the increases in expression of Drp1 and IL-1β and attenuated cognitive deficits caused by AβOs in mice. Interestingly, AβOs failed to alter hippocampal expression of mitochondrial proteins and to cause cognitive impairment in IL-1 receptor knockout mice (*Il1r1*^*-/-*^) mice, implicating an inflammatory mechanism in the impaired balance of mitochondrial fission/fusion proteins that is linked to synapse loss and cognitive impairment. Together, results provide a basis for targeting IL-1β signaling and the expression of mitochondrial fission and fusion proteins as a potential therapeutic approach for AD.

## Material and methods

### Reagents

Aβ_1-42_ was from American Peptide (Sunnyvale, CA, USA). Ham’s F12 medium (HF12-02) was from Caisson Labs (Smithfield, UT, USA). 1,1,1,3,3,3-Hexafluoro-2-propanol (HFIP), dimethyl sulfoxide (DMSO), bovine serum albumin (BSA), and mdivi-1 were from Sigma (St Louis, MO, USA). Prolong Gold Antifade, normal goat serum, and 4′,6-diamidino-2-phenylindole (DAPI) were from Invitrogen (Carlsbad, CA, USA). Commercial IL-1ra (anakinra, Kineret) was from Swedish Orphan Biovitrum (Stockholm, Sweeden). MitoSOX™ Red Mitochondrial Superoxide Indicator was from Thermo Fisher (Waltham, MA, USA).

### Aβ oligomers (AβOs)

AβOs were prepared from Aβ_1-42_ in aliquots as a dried hexafluoro-2-propanol film and stored at − 80 °C. The peptide film was dissolved in undiluted, sterile Me2SO to make a 5 mM solution. The solution was diluted to 100 μM with PBS medium without glutamine (BioSource International, Rockville, MD, USA) and aged overnight at 4 °C. The preparation was centrifuged at 14,000×*g* for 10 min at 4 °C to remove insoluble aggregates (protofibrils and fibrils), and the supernatants containing soluble AβOs were transferred to clean tubes and stored at 4 °C. AβOs were routinely prepared and characterized by size-exclusion chromatography (HPLC) and immunocytochemistry to detect oligomer binding to neurons with NU4 and absence of larger Aβ aggregates (Suppl. Fig 1) [42]. Biophysical/biochemical characterization of AβOs has also been described previously by our group [43–46]. For experiments with cynomolgus macaques, AβOs were prepared in cold Ham’s-F12 and routinely characterized by HPLC. AβOs were always kept in 4 °C and used within 24 h.

The dosages of AβOs used in vitro and in vivo experiments were based on previous studies from our group. We used a subtoxic concentration of AβOs in cultures [47]. The in vivo dosage was chosen to be the lowest dose capable of causing persistent (3-week) memory impairment in mice [44].

### Treatments of cultures or mice

Hippocampal cultures were exposed to 500 nM AβOs or an equivalent volume of vehicle (2% DMSO in phosphate-buffered saline (PBS) for 24 h). For IL-1 receptor blockade in hippocampal cultures, anakinra was added at a concentration of 100 ng/μL an hour before exposure to AβOs. Mitochondrial Division inhibitor 1 (Mdivi-1) (25 μM) was added to cultures 1 h before addition of AβOs. Dosage of mdivi-1 was based on previous in vitro studies using this inhibitor [13, 48]. For in vitro experiments with MitoSOX Red, a mitochondrial superoxide indicator, 2.5 μM was added to cultures for 10 min following the end of treatment with either AβOs, mdivi-1, or anakinra. For inhibition of mitochondrial division in vivo, mice received mdivi-1 (40 mg/kg) or an equal volume of saline (i.p.) for 7 days. The choice of dose used in vivo was based on a previous literature report [13] and on the distribution and pharmacokinetics of mdivi-1 in mice [49].

### Mature hippocampal cultures and immunocytochemistry

Mixed hippocampal cultures were used after 18–21 days in vitro. Primary rat hippocampal cultures were prepared and developed according to established procedure [50] and adapted from the original protocol described by Brewer [51]. Briefly, hippocampi from 18-day-old embryos were dissected and the meninges were removed. After that, tissue was dissociated in 0.05% PBS trypsin/EDTA solution at 37 °C for 5–10 min under occasional gentle shaking, followed by mechanical dissociation with glass Pasteur pipettes in DMEM medium supplemented with 5% horse-serum and 100 U/ml penicillin/streptomycin. Cells in suspension were counted in a Neubauer chamber and were plated on glass coverslips previously coated with 1 mg/ml poly-l-lysine, respectively. The density was 50.000 cells/well (260 cells/mm^2^) on coverslips for immunocytochemistry experiments. Cell cultures were maintained at 37 °C in a humidified atmosphere containing 5% CO2 for 60 min, and DMEM was replaced by Neurobasal plus medium supplemented with 2% B27 supplement, 0.5 mM Glutamax. Cultures were maintained at 37 °C in a humidified atmosphere containing 5% CO2 for 18–21 days prior to use. As shown in Suppl. Fig. 2a, we used mixed hippocampal neurons (containing ~ 40% neuronal cells and ~60% non-neuronal cells) in our study, to allow the cross talk between neuronal and non-neuronal cells.

All procedures were approved by the Institutional Animal Care and Use Committee of the Federal University of Rio de Janeiro (protocol #IBQM 022). After the pharmacological treatments described above, cultures were fixed with 4% paraformaldehyde for 15 min, permeabilized with 0.1% Triton X-100 for 5 min at room temperature, and blocked with 10% normal goat serum in PBS for 1 h before immunoreactions with anti-synaptophysin (mouse, 1:1,000; Abcam 14692), anti-postsynaptic density protein 5 (PSD-95) (rabbit, 1:500; Synaptic Systems 124 003), anti-MFN-1 (mouse, 1:200; Abcam Ab57602), or anti-MFN-2 (rabbit, 1:200; Sigma M6319). After overnight incubation with primary antibodies, cells were washed with PBS and incubated with Alexa-conjugated secondary antibodies for 2 h at room temperature. Nuclei were counterstained with ProLong containing DAPI antifade mounting solution. Coverslips were imaged on a Nikon C2 confocal microscope. MFN-1 and MFN-2 immunofluorescence intensities were analyzed in at least 3–4 experiments each (see figure legends) using independent neuronal cultures and AβO preparations. In each experiment, 20–30 images (from 2 to 3 coverslips) were acquired from each experimental condition. Histogram analysis of fluorescence at each pixel across the images was performed using FIJI (National Institutes of Health), as described previously [52]. For quantification of synaptic punctae, a total of 15–20 images per experimental condition were acquired. PSD-95 (red) and synaptophysin (green) immunofluorescent puncta were analyzed and quantified using the Puncta Analyzer plugin in NIH FIJI as previously described [43, 53, 54].

### Animals

Male C57BL/6 and *Il1r1*^*−/−*^ (on a C57BL/6 background) mice, originally obtained from The Jackson Laboratories, were obtained from the animal facility at the Federal University of Rio de Janeiro and were 3 months old at the beginning of experiments. All procedures were approved by the Federal University of Rio de Janeiro Animal Care Committee (protocol number 022/18) and were in full compliance with the NIH Guide for Care and Use of Laboratory. All mice in this study were housed five per cage with ad libitum access to food and water in temperature- and humidity-controlled rooms on a 12-h light/dark cycle. The studies performed with macaques were approved by the Queen’s University Animal Care Committee and were in full compliance with the Canada Council on Animal Care (Animal Care Protocol Original Munoz, 2011-039-Or). All macaques were maintained at the Centre for Neuroscience Studies at Queen’s University (Kingston, Canada) and closely monitored by a lab animal technician and the Institute’s veterinarian.

### Brain infusion of AβOs in mice and treatment with mdivi-1

For i.c.v. infusion of AβOs, mice were anesthetized for 7 min with 2.5% isoflurane (Cristália, São Paulo, Brazil) using a vaporizer system and gently restrained during the injection procedure. A final volume of 3 μl (100 pmol AβOs or vehicle) was injected into the lateral ventricle of the mice as described previously [44, 53, 55–57]. Briefly, a 2.5-mm needle was inserted unilaterally 1 mm to the right of the midline point equidistant from each eye and 1 mm posterior to a line drawn through the anterior base of the eye. Mice that showed signs of misplaced injections or any sign of hemorrhage (5% of cases, on average) were excluded from statistical analysis. For inhibition of mitochondrial fission, mice received daily i.p. injections of mdivi-1 (40 mg/Kg) or vehicle (PBS) for 7 days. Dosage of mdivi-1 was based on previous in vivo studies using this inhibitor [10, 13, 58, 59].

### RNA extraction and qPCR

Hippocampi were homogenized in 1 ml Trizol (Thermo Fisher Scientific), and RNA extraction was performed using the SV Total RNA Isolation System (Promega) according to the manufacturer’s instructions. Purity and amount of RNA were determined by the 260/280 nm absorbance ratio. Only preparations with ratios between 2.2 and 1.8 and no signs of RNA degradation were used. One microgram RNA was used for cDNA synthesis using the High-Capacity cDNA Reverse Transcription Kit (Thermo Fisher Scientific). Expression of genes of interest was analyzed by qPCR on an Applied Biosystems 7500 RT-PCR system using the Power SYBR kit (Applied Biosystems, Waltham, MA, USA). Actin was used as an endogenous control. Cycle threshold (Ct) values were used to calculate fold-changes in gene expression using the 2^−ΔΔCt^ method [60]. In all cases, reactions were performed in 15 μl. Primer sequences for genes studied were: MFN-1, Forward: 5′-TCTCCAAGCCCAACATCTTCA-3′ and Reverse: 5′-ACTCCGGCTCCGAAGCA-3′; MFN-2, Forward: 5′-GGGGCCTACATCCAAGAGAG-3′ and Reverse: 5′-GGAGAACTTTGTCCCAGAGC-3′; OPA-1, Forward: 5′-TGGGCTGCAGAGGATGG-3′ and Reverse: 5′-CCTGATGTCACGGTGTTGAT-3′; Drp1, Forward: 5′-AGACGCTTAATCTGACGTTTGAC-3′ and Reverse: 5′-AGGTGGCCTTAACACTATTGACA-3′; Actb, Forward: 5′-TGTGACGTTGACATCCGTAAA-3′ and Reverse: 5′-GTACTTGCGCTCAGGAGGAG-3′.

### Brain infusion of AβOs in macaques

Seven female cynomolgus macaques (*Maccaca fascicularis*, body weights: 4.7–7.0 kg) were used. Three sham-operated macaques, 9 years of age (*n* = 1) and 16 years of age (*n* = 2), were used as controls and underwent the full surgical procedure but did not receive intracerebroventricular injections. After a recovery period of 2–4 weeks, four macaques, 9 years old (*n* = 2) and 16 years old (*n* = 2), had a guide cannula inserted into the lateral ventricle. Control animals had the cannula implanted into the lateral ventricle in the same manner as the experimental animals but did not receive oligomer injections. A total of 4 animals received 10–100 μg i.c.v. of AβOs (1 injection per day every 3 days for up to 24 days) while sedated using ketamine (Vetoquinol, Québec, ON, Canada; 3mg/kg), medetomidine (Sandoz, Québec, ON, Canada; 0.15 mg/kg), and glycopyrrolate (Sandoz; 0.013 mg/kg). Although the experimental design involved injection of a fixed amount of 100 μg of AβOs per injection in all macaques, this amount was somewhat variable among animals, due to procedural limitations at the moment of injection, due to procedural limitations at the moment of injection, including partial clogging of the cannulas and liquid reflux through the cannula following injection. The total amount of AβOs effectively introduced into the lateral ventricle in each animal was 300 μg (macaque 1), 560 μg (macaque 2), 544 μg (macaque 3), and 474 μg (macaque 4), as described in detail in our previous study [61]. Approximately 1 week after completion of the experimental protocol, macaques were sedated with intramuscular ketamine (10 mg/kg) followed by intravenous sodium pentobarbital (25 mg/kg) and heparin. They were then perfused intracardially with PBS followed by 4% paraformaldehyde in PBS. Brains were removed, and coronal 40-μm-thick sections were obtained.

### Immunohistochemistry in macaque brain sections

For immunofluorescence analysis of macaque brain sections, tissue autofluorescence was quenched by incubation with 0.06% potassium permanganate (Merck) for 10 min at room temperature. Sections were blocked in 5% BSA and 5% normal goat serum in 1% Triton X-100 for 3 h at room temperature. Primary antibodies anti-MFN-1 (mouse, 1:200; Abcam Ab57602), MFN-2 (rabbit, 1:200; Sigma, M6319), OPA-1 (rabbit, 1:500; Abcam Ab42364), and Drp1 (rabbit, 1:100; Novus Biological NB11055237) were diluted in blocking solution, and sections were incubated at 4 °C for 16 h, followed by incubation with Alexa594-conjugated secondary antibodies (donkey anti-goat, 1:2,000; Thermo Fisher Scientific R37119) for 2 h at room temperature. Nuclei were stained with DAPI (Invitrogen, Carlsbad, CA, USA) for 5 min. Immunostaining was carried out in parallel in brain sections from all animals, and constant parameters for image acquisition and analysis were used. Slides were mounted with Prolong Gold Antifade with DAPI (Invitrogen) and imaged on a Zeiss Axio Observer Z1 microscope equipped with an Apotome module to minimize out-of-focus light. Cells positive for MFN-1, MFN-2, and Drp1 were manually counted in 20 z-stack images acquired using the same parameters from each region of interest for each animal. For quantification of OPA-1, a total of 15–20 z-stack images from each region of interest for each animal were acquired with the same parameters. The number of puncta was analyzed and quantified using the Puncta Analyzer plugin in NIH FIJI.

### Behavioral testing

#### Novel object recognition (NOR)

The novel object recognition (NOR) memory task was carried out in an open field arena measuring 0.3 × 0.3 × 0.45 m as described [44]. Test objects were made of glass or plastic and had different shapes, colors, sizes, and textures. During both training and test sessions, objects were taped to the floor of the arena so that the animals could not move them. None of the objects used in the current study evoked innate preference. Initially, animals were trained in a 5-min habituation session, in which they were allowed to freely explore the empty arena. Total distance and velocity during the 5-min open field session were recorded as measures of locomotor activity, and no differences were found among experimental groups. After that, animals were placed in the arena for the training session, in which animals were placed at the center of the arena in the presence of two identical objects. The amount of time spent exploring each object was recorded. After 1 h, animals were again placed in the arena for the test session, when one of the familiar objects was replaced by a different (novel) object. Results are expressed as the percentage of time exploring each object during the training and test sessions. Animals with normal memory remember the familiar object as such and spend more time exploring the novel object in the test session.

#### Contextual fear conditioning (CFC)

Contextual fear conditioning (CFC) was evaluated as described [53, 55, 56]. The apparatus (25 × 25 × 25 cm) consisted of a box with aluminum walls with a methacrylate door and a grid floor composed of stainless-steel bars spaced 1 cm apart and connected to an electric shock generator (Panlab®, Harvard Apparatus, Cornellà, Spain). Each mouse was placed in this chamber and allowed to freely explore the conditioning box for 3 min before receiving 2 footshocks (0.35 mA for 2 s, with 30 s interval between shocks). Each mouse remained in the chamber for 30 s after the last shock before being returned to its home cage. After 24 h, animals were again placed in the conditioning chamber for 5 min and freezing behavior was quantified using the Freezing® software version 1.3.04 (Panlab).

### Statistical analysis

Results are presented as means ± SEM. All statistical analyses were performed using GraphPad Prism (version 8.0). Student’s *t* test or two-way ANOVA followed by Holm-Šidák post hoc test was used for group comparisons, as indicated in the figure legends. Data from the NOR task were analyzed using a one-sample *t* test, comparing time spent exploring the novel object to the fixed value of 50% (chance level) as previously described [44]. *p* values < 0.05 were considered statistically significant.

## Results

### Anakinra, an IL-1 receptor antagonist, attenuates the impact of AβOs on mitochondrial membrane potential and fusion/fission proteins

IL-1β has been found to be elevated in human AD brains [30–32] and in the brains of AβO-infused mice [34]. IL-1β has also been implicated in mitochondrial dysfunction and damage [25, 62–64]. Thus, we initially investigated whether AβO could induce alterations in mitochondrial fusion/fission proteins and whether activation of pro-inflammatory IL-1β signaling could be involved in mitochondrial pathology in primary hippocampal mixed cultures. Characterization of AβOs by HPLC shows the presence of low- and high-molecular oligomers (Suppl. Fig. 1). Immunocytochemistry experiments further reveal AβO binding to neurons and the absence of larger aggregated species (Suppl. Fig. 2b).

**Fig. 1:**
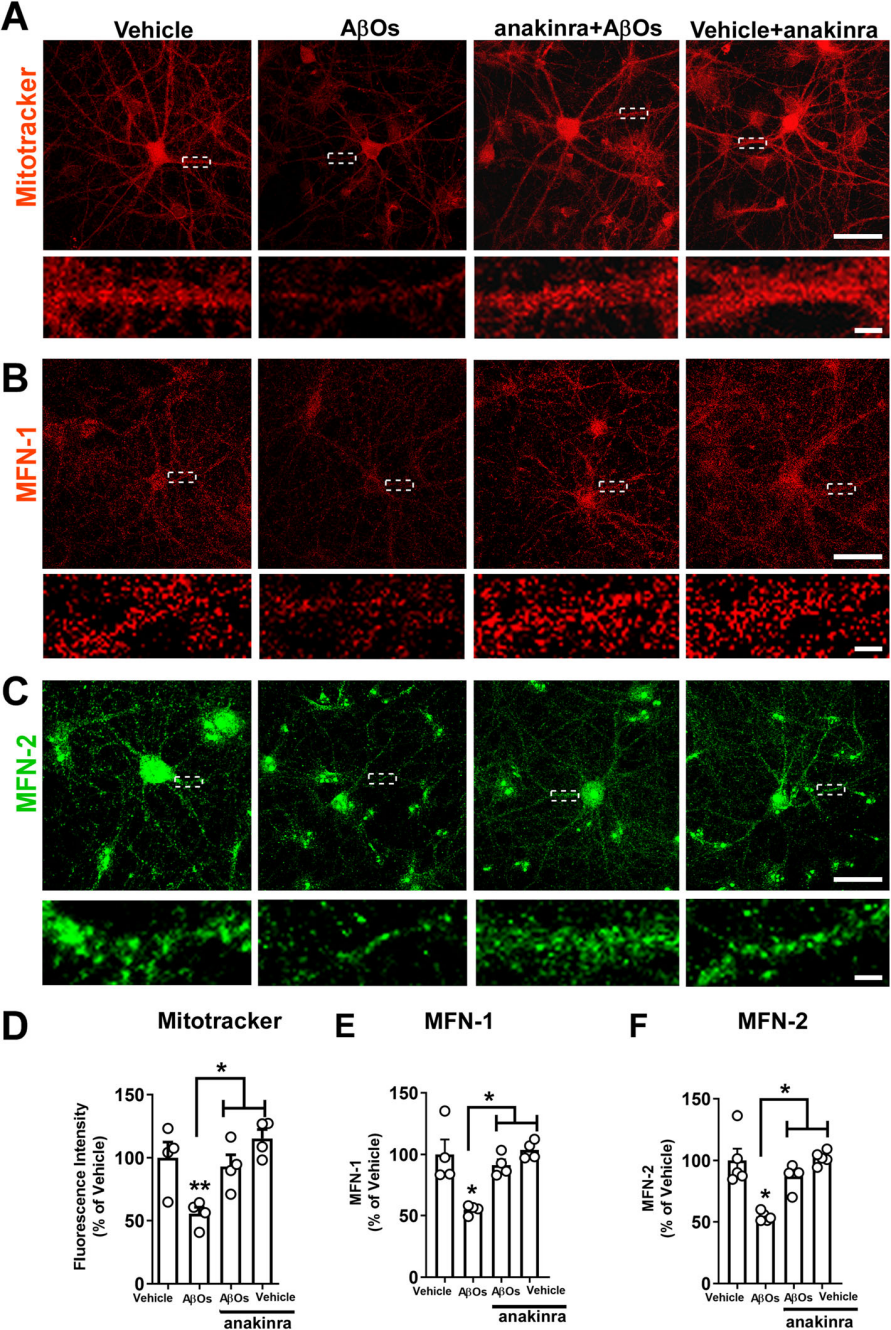
IL-1 antagonist alleviates AβO-induced loss of mitochondrial membrane potential and fusion proteins in mature hippocampal cultures. **a** Representative images of mature hippocampal cultures exposed to AβOs (500 nM) or vehicle for 24 h. Where indicated, primary cultures were pre-treated for 40 min with anakinra (100 μM). The cultures were stained with a selective mitochondrial probe, Mitotracker (100 nM) (red). **b**, **c** Representative images of mature hippocampal cultures immunolabeled for MFN-1 (red) and MFN-2 (green). Scale bar: 60 μm. Optical zoom images of selected regions are also presented. Scale bar: 5 μm**. d** Integrated immunofluorescence of mitotracker (100 nM). Data expressed as means ± SEM from 4 experiments with independent cultures (30 images analyzed per experimental condition per experiment). *p < 0.05, **p < 0.01 compared with vehicle or AβOs; Two-way ANOVA followed by Holm-Šidak post hoc test. **e**, **f** Integrated immunoreactivities for MFN-1 and MFN-2. Data are expressed as means ± SEM from 4 to 5 experiments with independent cultures (30 images analyzed per experimental condition per experiment), *p < 0.05 compared with vehicle or AβOs. Two-way ANOVA followed by Holm-Šidak post hoc test

We first used anakinra, a recombinant form of the human endogenous IL-1 receptor antagonist (IL-1ra), which has been used in the treatment of rheumatoid arthritis [36, 65]. Treatment with anakinra prevented the decrease in mitochondrial membrane potential induced by AβOs in hippocampal neurons (Fig. 1a, d). Moreover, anakinra attenuated the reductions in MFN-1 (Fig. 1b, e) and MFN-2 (Fig. 1c, f) immunoreactivities in AβO-treated hippocampal neurons. These results suggest that IL-1 receptor activation induces mitochondrial dysfunction in neurons exposed to AβOs.

### Aβ oligomers alter levels of mitochondrial fusion/fission proteins in the brains of cynomolgus macaques

**Fig. 2:**
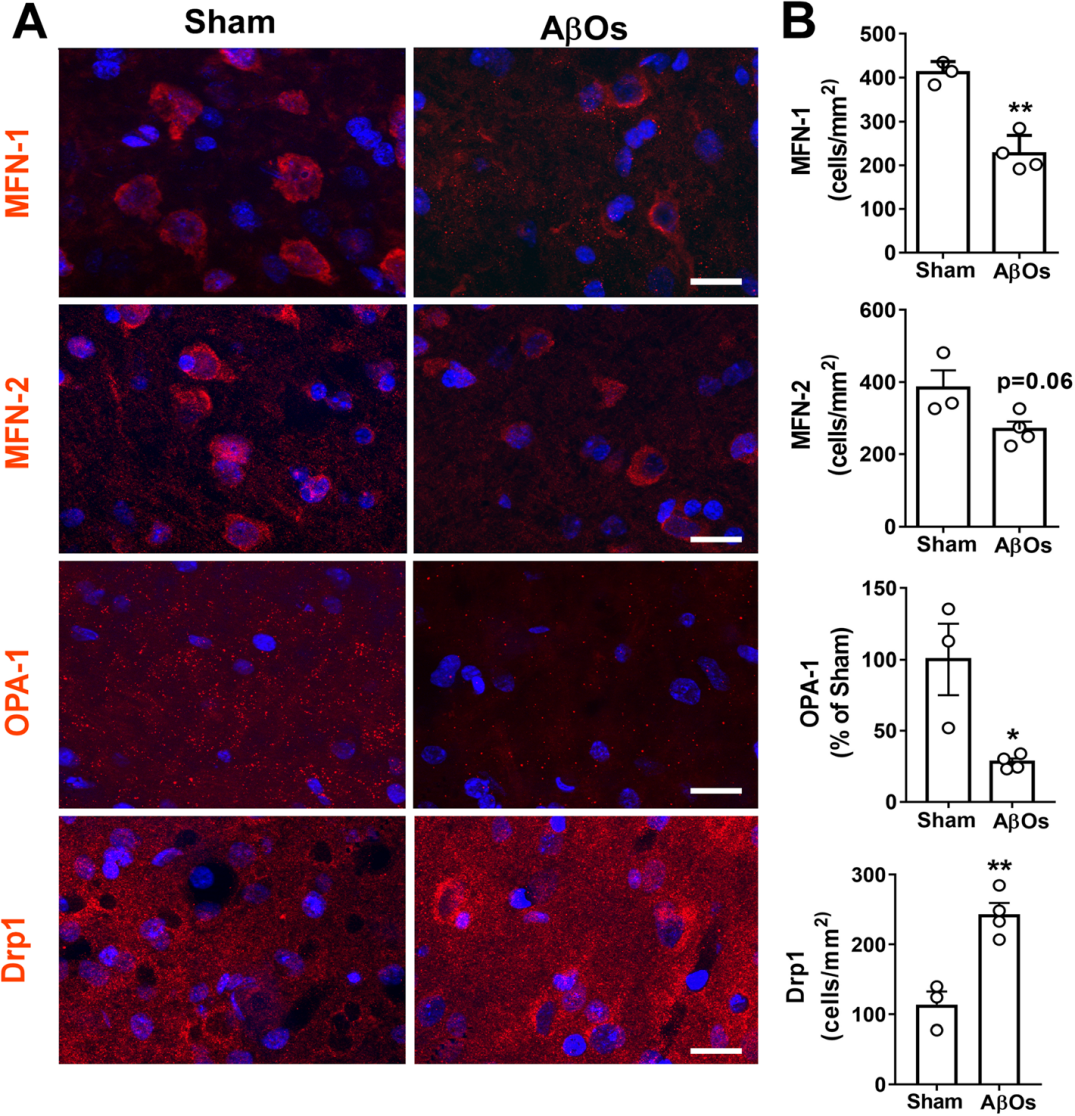
AβOs trigger abnormal mitochondrial dynamic proteins changes in the brains of cynomolgus macaques. **a** Representative images of MFN-1, MFN-2, OPA-1, and Drp1 immunolabeling in the frontal cortex of sham-operated and AβO-injected macaques. Nuclear staining (DAPI) is shown in blue. Scale bar: 20 μM. **b** Quantification of MFN-1-, MFN-2-, OPA-1-, and Drp1-positive cells. Data are expressed as means ± SEM (*n* = 3 sham; *n* = 4 AβOs). **p < 0.01, *p < 0.05, Student’s unpaired *t* test

We tested whether AβOs, implicated as proximal synaptotoxins in AD [2, 20], might alter mitochondrial fission/fusion proteins in the primate brain. To this end, we carried out i.c.v. infusions of AβOs in cynomolgus macaques (*Macaca fascicularis*), a model that recapitulates several aspects of human AD pathology, including tau hyperphosphorylation, tangle formation, synapse loss, and brain inflammation [53, 56, 61, 66]. We investigated the levels of mitochondrial proteins in the frontal cortex of macaques that received i.c.v. AβO infusions because our previous study indicated that this region is targeted by AβOs [61]. In accordance, we observed AβO labeling in the frontal cortex of macaques that received AβO infusions, while no labeling was observed in control macaques (Suppl. Fig. 2c). AβOs caused decreases in densities of MFN-1- and MFN-2-positive cells and in the number of OPA-1 immunoreactive puncta and an increase in the number of Drp1-positive cells in the frontal cortex of macaques (Fig. 2a, b). These results are consistent with alterations in these proteins reported in post-mortem AD brains [12, 18] and suggest that AβOs may underlie the alterations of levels of mitochondrial fusion/fission proteins in the AD brain.

### Mdivi-1 prevents AβO-induced synapse loss and rescues memory impairment in mice

**Fig. 3:**
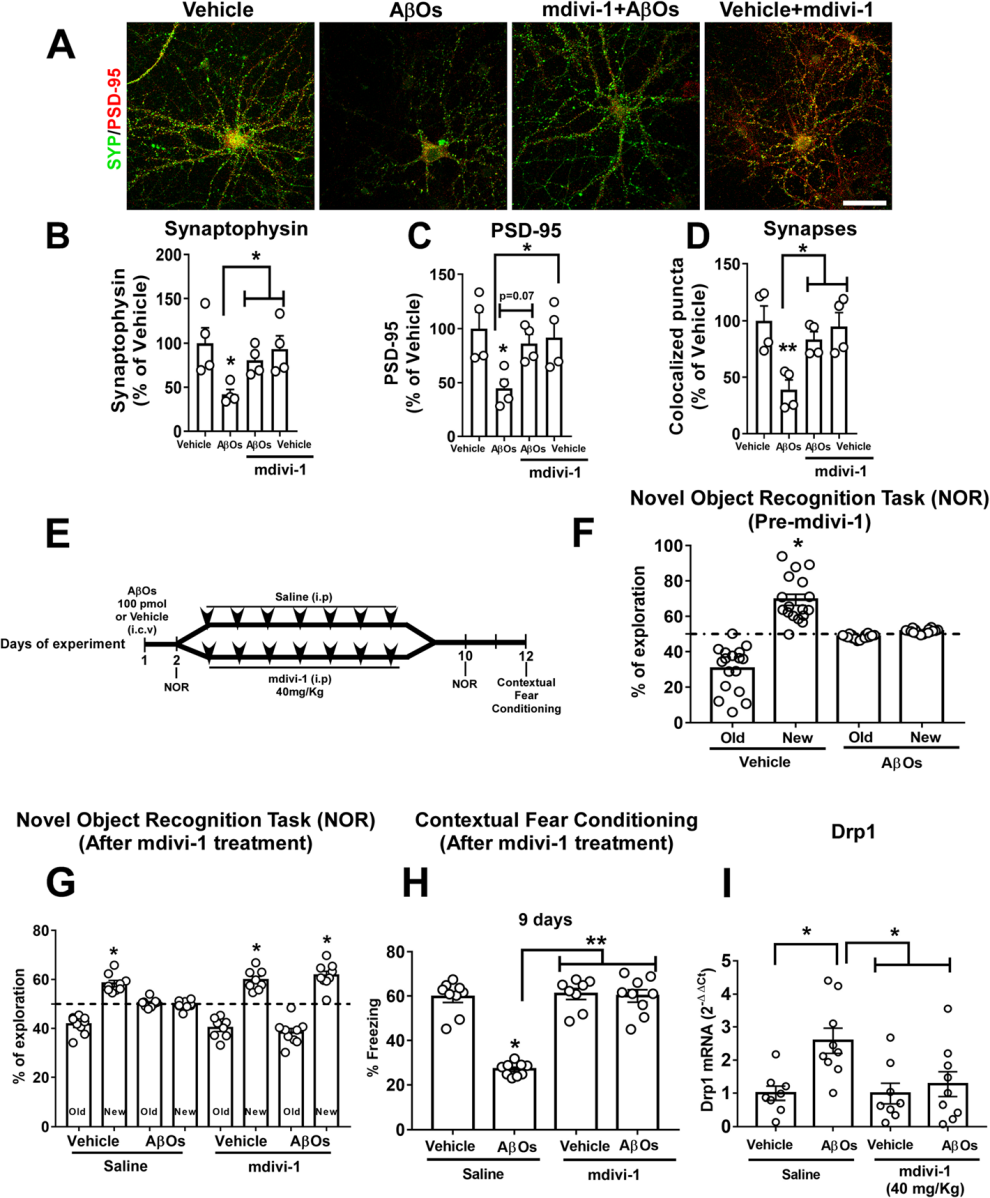
Mdivi-1 rescues AβO-induced cognitive impairment, defective mitochondrial fission protein expression, and inflammation in mice and prevents synapse loss in hippocampal neurons. **a** Representative images of cultured hippocampal neurons exposed to 500 nM AβOs or vehicle for 24 h and immunolabeled for synaptophysin (green) or PSD-95 (red). Synapses evidenced by colocalized puncta, appear in yellow. Where indicated, neurons were pre-incubated for 1 h with mdivi-1 (25 μM). Scale bar: 20 μm. **b**, **d** Integrated immunoreactivities, for synaptophysin (**b**), PSD-95 (**c**), and number of colocalized synaptophysin/PSD95 puncta (**d**). Data are expressed as means ± SEM from 4 experiments from independent cultures (30 images analyzed per experimental condition per independent culture). *p < 0.05, **p < 0.01 compared with vehicle or AβOs; two-way ANOVA followed by Holm-Šidak post hoc test. **e** Experimental design of the treatment of mdivi-1. Male C57BL/6 mice received an i.c.v. injection of AβOs (100 pmol) or vehicle. Twenty-four hours after i.c.v. injection of AβOs (100 pmol) or vehicle, animals underwent the NOR. The percentage of time exploring familiar or novel objects in the test session is shown in **f**. Mdivi-1 (40 mg/kg, i.p.) treatment began on day 3 and lasted for 7 days. On day 10, animals underwent NOR again. **g** after the mdivi-1 (40 mg/kg, i.p.) treatment. **h** 11 days after i.c.v. injection of AβOs (100 pmol), the same animals were trained in the contextual fear conditioning task. The percentage of freezing behavior in this task is shown. In **f** (*n* = 17–18 animals per group) and **g** (*n* = 9–10 animals per group), data are expressed as means ± SEM of the percentage of time spent on each object presented at the test session and *p < 0.05, compared with a hypothetical value of 50% of exploration time; one-sample Student’s *t* test. In **h**, data are expressed as means ± SEM (*n* = 9–10 animals per group). **p < 0.01, *p < 0.05, compared with vehicle or AβOs; two-way ANOVA followed by Holm-Šidak post hoc test. **i** Drp1 levels in the hippocampi of mice 12 days after i.c.v. injection of AβOs (100 pmol). Following AβO injection, animals were treated for 7 days with saline or mdivi-1 (40 mg/kg; i.p.; *n* = 8-9 animals/group). *p < 0.05 compared with vehicle or AβOs, two-way ANOVA followed by Holm-Šidak post hoc test

Mitochondrial fission is upregulated in AD, and inhibitors of fission have been proposed as a strategy to counteract deregulated mitochondrial dynamics in AD [67–69]. This prompted us to evaluate whether mdivi-1, an inhibitor of Drp1, would be effective against the deleterious effects of AβOs. In hippocampal cultures, treatment with mdivi-1 attenuated the decreases in pre- and post-synaptic markers (synaptophysin, shown in green, and PSD-95, shown in red, respectively) and synapse loss induced by exposure to AβOs (Fig. 3a–d). Results suggest that impaired mitochondrial dynamics contribute to synapse loss induced by AβOs.

Next, we determined whether mdivi-1 could prevent memory deficits induced by AβOs in mice. To this end, mice received an i.c.v. infusion of AβOs and were assessed in the novel object recognition (NOR) test. Mice were then separated into two groups that were treated with saline or mdivi-1 (40 mg/Kg, i.p.) for 7 days (Fig. 3e) before re-testing in the NOR test. Consistent with our previous reports [43, 44, 53, 55, 56], i.c.v infusion of AβOs caused an impairment in object recognition memory 24-h post-infusion (Fig. 3f). While memory impairment persisted in saline-treated animals, AβO-infused mice that were treated with mdivi-1 showed normal performance in the NOR task (Fig. 3g). The same mice were further assessed in the contextual fear conditioning (CFC) task, a hippocampus- and amygdala-dependent test [70]. Twenty-four hours after the training session, AβO-infused mice presented significantly less freezing behavior compared to vehicle-injected mice, indicating impaired contextual memory (Fig. 3h). In contrast, AβO-infused mice that were treated with mdivi-1 performed similar to control animals (Fig. 3h).

We further examined the effects of mdivi-1 on Drp1 expression in the hippocampi of mice that received an i.c.v. infusion of AβOs. While AβOs induced an increase in hippocampal expression of Drp1, treatment with mdivi-1 corrected Drp1 expression in AβO-infused mice (Fig. 3i). Results demonstrate that mdivi-1 rescued abnormal hippocampal expression of Drp1 and memory deficits induced by AβOs in mice.

### IL-1R mediates AβO-induced altered expression of mitochondrial fusion/fission proteins and memory impairment in mice

**Fig. 4:**
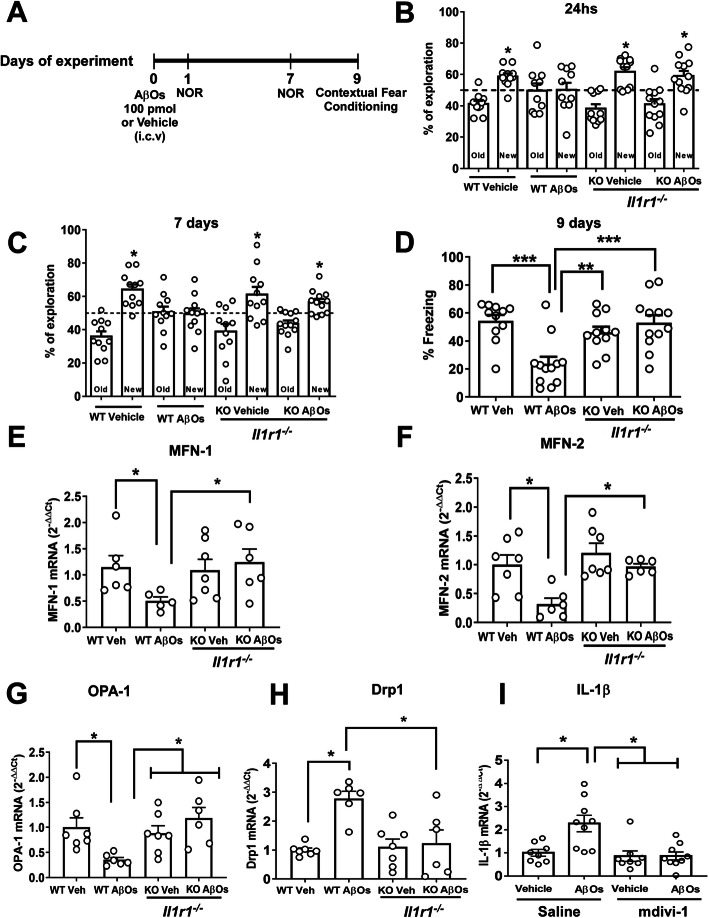
IL-1 signaling mediates cognitive impairment and abnormal expression of mitochondrial fission and fusion proteins caused by AβOs in mice. **a** Experimental design for behavior experiments with *Il1r1*^*−/−*^ mice i.c.v. injected with vehicle or 100 pmol AβOs. **b**, **c** Exploration times of WT or *Il1r1*^*−/−*^ mice 24 h or 7 days after a single i.c.v. injection with vehicle or 100 pmol AβOs in the NOR task. Data are expressed as means ± SEM (*n* = 11–12 animals per experimental group). Asterisks denote a statistically significant (*p* < 0.05) difference from 50% (reference value). One-sample Student’s *t* test. **d** 9 days after i.c.v. injection of AβOs (100 pmol), the same animals were trained in the contextual fear conditioning task. The percentage of freezing behavior in this task is shown. Data are expressed as means ± SEM (*n* = 6–7 animals per experimental group. **p < 0.01, ***p < 0.001 compared to vehicle or AβOs, two-way ANOVA followed by Holm-Šidak post hoc test. **e** MFN-1, **f** MFN-2, **g** OPA-1, and **h** Drp1 mRNA levels in the hippocampi of mice 12 days after i.c.v. injection of AβOs (100 pmol). *p < 0.05 compared with vehicle or AβOs (*n* = 6–7 animals per experimental group). Two-way ANOVA followed by Holm-Šidak post hoc test. **i** IL-1β mRNA levels in the hippocampi of mice 12 days after i.c.v. injection of AβOs (100 pmol). Following AβO injection, animals were treated for 7 days with saline or mdivi-1 (40 mg/kg; i.p.; *n* = 9–10 animals/group). *p < 0.05 compared with vehicle or AβOs, two-way ANOVA followed by Holm-Šidak post hoc test

While i.c.v. infusion of AβOs induced NOR memory impairment in wild-type (WT) mice, AβOs failed to impact memory in *Il1r1*^*−/−*^ mice either 24 h or 7 days post-infusion (Fig. 4a–c). Similar results were observed when memory was assessed in the CFC task 9 days after i.c.v infusion of AβOs (Fig. 4d). *Il1r1*^*−/−*^ mice were further protected against reductions in hippocampal expression of MFN-1, MFN-2, and OPA-1 (Fig. 4e–g) and increased expression of Drp1 (Fig. 4h) induced by AβOs. Control measurements showed no significant changes in overall mitochondrial content, as indicated by expression of COX-IV, between vehicle- and AβO-infused mice (Suppl. Fig. 3).

Finally, because previous studies have shown that excessive mitochondrial fission exacerbates the production of pro-inflammatory mediators by microglial cells [64, 71–74], we examined whether inhibition of Drp1 would alter IL-1β expression in mice. Whereas AβOs induced increased hippocampal expression of IL-1β in control (saline-treated) mice, AβOs failed to increase IL-1β expression in the hippocampi of mdivi-1-treated mice (Fig. 4i). Results thus indicate that treatment with mdivi-1 decreases hippocampal IL-1β expression in mice and link pro-inflammatory IL-1β signaling to impairments in mitochondrial dynamics.

## Discussion

In the current work, we show that AβOs, toxins that accumulate in AD brains [75, 76] and have been implicated in synapse damage and memory impairment in AD [2, 20], trigger alterations in proteins involved in mitochondrial dynamics in experimental models of AD, including cynomolgus macaques that received i.c.v. injections of AβOs. Changes in mitochondrial proteins and cognitive impairment induced by AβOs were found to be mediated by IL-1R activation.

Previous studies have reported changes in proteins involved in mitochondrial fission/fusion in vitro and in AD mouse models [6, 9, 10, 13, 19, 77]. In post-mortem AD brains, decreased expression of fusion proteins MFN-1, MFN-2, and OPA-1 and increased expression of fission proteins Fis-1 and Drp1 have been reported [12, 19]. These findings suggest that an imbalance in levels of proteins involved in mitochondrial dynamics play a role in the pathogenesis of AD.

Aβ has been found to induce decreased expression of fusion proteins (MFN-1, MFN-2, and OPA-1) and increased expression of fission proteins (Drp1 and Fis-1) in AD models [3, 12, 18, 67, 78]. Recent studies further showed that excessive mitochondrial fission caused by increased Drp1 expression led to mitochondrial dysfunction in neurons exposed to Aβ [13, 67]. While our findings are in line with those studies, it is important to note that Aβ has been proposed to induce pathology by inhibiting Drp1 or fission more generally [77, 79, 80]. In addition, decreases in mitochondrial fission have been shown to cause fragmentation and mitochondrial abnormalities. Such studies suggest that any imbalance of fusion/fission could be detrimental and future studies aiming at investigating the role of Drp1 in health and disease are warranted. While Drp1 inhibition may be detrimental in physiological conditions, it is possible that pathological conditions that cause increases in Drp1 levels interfere with mechanisms involved in memory formation.

Mitochondria play a vital role in maintaining cellular function through their capacity to produce energy [81]. Fusion and fission mechanisms participate in the regulation of mitochondrial function, and disruption of these processes alters cellular localization and function of these organelles. Mitochondrial dynamics is physiologically relevant in regulation of mitochondrial function and metabolism, apoptosis, calcium homeostasis, autophagy, and mitophagy [5, 82]. Altered expression of proteins involved in mitochondrial dynamics has been associated with aging and age-related alterations [6, 10]. We found that alterations in mitochondrial fusion/fission proteins induced by AβOs are linked to synapse damage/loss and memory impairment. Mitochondrial dysfunction has been associated with synaptic failure [7, 9, 13, 67]. In neurons, mitochondrial dynamics has been implicated in the formation of synapses and dendritic spines [6, 7, 81]. GTPases MFN-1, MFN-2, and OPA-1 appear to be key for mitochondrial fusion, whereas Drp1 is needed for fission [6]. Mdivi-1 is expected to inhibit Drp1 and induce several outcomes that impact cell physiology. It is noteworthy the findings suggesting that mdivi-1 is not a specific Drp1 inhibitor, as mdivi-1 was found to modulate ROS generation by brain mitochondria, reversibly inhibit complex I in WT and Drp1 knockout (KO) fibroblasts [37]. In addition, in cancer cells, mdivi-1 was shown to decrease oxidative metabolism [41]. In the yeast pathogen Candida albicans, mdivi-1 was shown to impact endogenous nitric oxide levels [39]. Collectively, these studies indicate that such actions of mdivi-1 may contribute to its effects reported in our study and in other models of disease [38, 40]. Future studies will be important to better understand the role of mdivi-1 in physiological and pathological conditions.

Brain inflammation plays a key role in neuronal dysfunction in AD [23, 83]. Levels of IL-1β have been shown to be elevated in AD brains [30, 84]. Moreover, studies have suggested that neuroinflammation triggered by IL-1β impairs mitochondrial function [25, 62, 63]. Anakinra, a recombinant IL-1R antagonist [36, 65], prevented AβO-induced mitochondrial dysfunction and alterations in MFN-1 and MFN-2 levels in mixed hippocampal cultures. AβO-induced alterations in expression of mitochondrial fusion/fission proteins and cognitive impairment in mice were found to depend on IL-1R activation. Aβ is thus likely impacting glial cells that are present in hippocampal cultures and such cells are expected to play a key role in the detrimental effects of Aβ on mitochondrial proteins. Our findings implicate altered mitochondrial dynamics in IL-1β-mediated synaptic and memory defects and are consistent with studies showing that blockade of IL-1R restores cognition and alleviates pathogenesis in AD mouse models [26, 27]. While the role of neuroinflammation on neuronal mitochondrial dynamics in AD has not been completely explored, there is evidence showing that production of inflammatory mediators can impair mitochondrial function [63, 85].

A very recent study [63] showed that overexpression of MFN-2 prevented peripheral lipopolysaccharide (LPS)-induced neuroinflammation in mice, with significant reductions in brain IL-1β levels and mitochondrial fragmentation in neurons. Collectively, these data support our hypothesis that neuroinflammation mediated by IL-1β affects mitochondrial dynamics and memory impairment in mice.

Interestingly, decreases in MFN-1 and MFN-2 in the brain were reported in a mouse model of metabolic syndrome that presented microglial activation and increased IL-1β expression [86]. Fang and colleagues (2019) further reported that Aβ inhibited mitophagy and that improvements in neuronal mitophagy enhanced the phagocytic efficiency of microglia and decreased pro-inflammatory cytokines, including IL-1β [87]. These effects were associated with reversion of memory impairment in mice.

Restoration of mitochondrial fission/fusion balance has been proposed as a therapeutic strategy in neurodegenerative disorders, including Alzheimer’s, Hungtinton’s, and Parkinson’s diseases and in metabolic disorders (i.e., diabetes and obesity) [13, 67–69, 78]. Baek and colleagues (2017) demonstrated that inhibition of Drp1 by mdivi-1 alleviated mitochondrial fragmentation, loss of mitochondrial membrane potential, and ROS production and prevented memory impairment in the APP/PS1 AD mouse model [13]. Consistent with these findings, we observed that inhibition of mitochondrial fission alleviated mitochondrial dysfunction and, notably, reversed memory deficits in AβO-infused mice.

Mitochondria are major players in the innate immune response [88, 89]. Studies have demonstrated the role of mitochondria in inflammation through activation of Nod-like receptor family, pyrin domain containing 3 (NLRP3) inflammasome [90, 91]. NLRP3 detects damaged mitochondria and initiates an inflammatory response leading to the release of IL-1β [91, 92]. Our findings extend those of previous studies in models of blood-brain barrier (BBB) disruption [72] and LPS-stimulated microglial cells [63, 64, 71], where treatment with mdivi-1 decreased levels of IL-1β and other pro-inflammatory mediators. Even though the acute effects of mdivi-1 on IL-1β have not been investigated in our study, our experimental design allowed the determination of IL-1β expression in mice that received an i.c.v. infusion of AβOs and were treated for 7 consecutive days with mdivi-1. Our findings that mdivi-1 blocks the increase in IL-1β expression induced by AβOs under these conditions suggests that blocking mitochondrial pathology may be relevant to block the sustained induction of IL-1β in mice. Fan and colleagues [72] reported that treatment with mdivi-1 decreased brain expression of IL-1β, TNF-ɑ, and IL-6 24 h after subarachnoid hemorrhage injury in rats. This suggests that, in addition to the longer-term effects revealed in our study, mdivi-1 may also have an acute impact on IL1-β in rodents. Our results further suggest that regulation of proper mitochondrial dynamics may comprise an approach to counteract excessive brain inflammation promoted by IL-1β. Interestingly, we found that AβOs failed to cause cognitive impairment in *Il1r1*^*−/−*^ mice and that anakinra attenuated alterations in the levels of mitochondrial proteins induced by AβOs in vitro. Our results thus implicate IL-1β-dependent signaling in the mechanism of mitochondrial dysfunction in AD experimental models.

## Conclusion

**Fig. 5:**
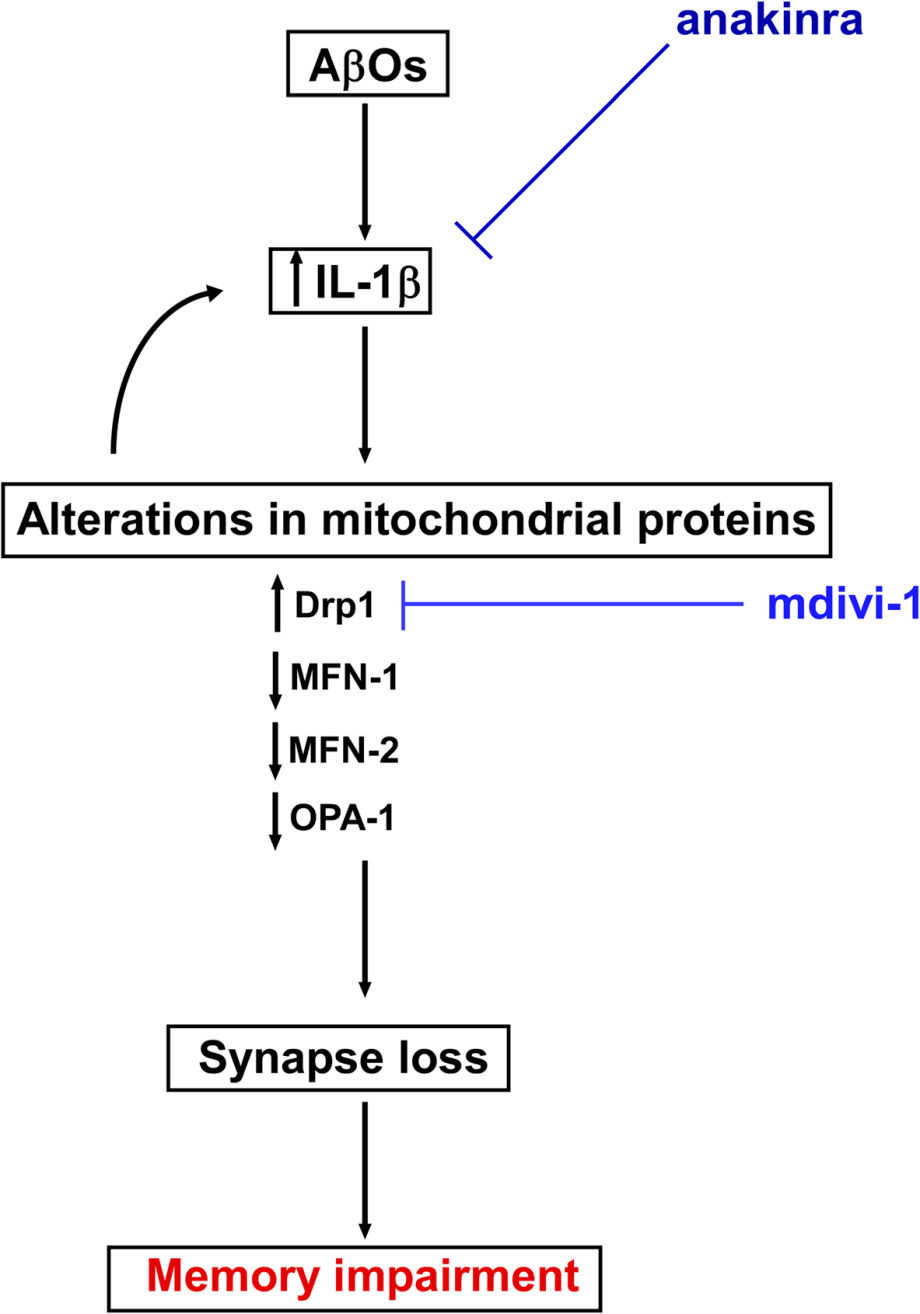
Schematic representation of the effects of AβOs inducing synapse loss, memory impairment, and abnormal mitochondrial dysfunction via IL-1β signaling. AβOs lead to increase brain levels of IL-1β, leading to perturbations in mitochondrial proteins, including an increase of Drp1 levels and decrease of MFN-1, MFN-2, and OPA-1, causing synapse loss and memory impairment. Anakinra, an IL-1 antagonist, decreases IL-1β expression leading to restoration of normal levels of mitochondrial proteins. Mdivi-1, a fission inhibitor, inhibits Drp1 increased, as well reduced IL-1β expression, suggesting a negative feedback loop between mitochondrial dynamics proteins and IL-1β activity

Taken together, our findings indicate that activation of IL-1 receptor mediates the alteration in levels of mitochondrial fission/fusion proteins, leading to memory impairment induced by AβOs (Fig. 5). Moreover, IL-1β expression in the hippocampus was found to be reduced following mdivi-1 treatment, suggesting a negative feedback loop between mitochondrial dynamics and IL-1β-mediated inflammation (Fig. 5). In conclusion, our results point to a bidirectional link between brain mitochondrial dynamics and IL-1β and suggest that strategies aimed to neutralize excessive IL-1β signaling may be interesting therapeutic approaches to protect mitochondrial dynamics, synapses and memory in AD.

## Supplementary Information

**Additional file 1.** Supplemental Figure 1. Characterization of AβOs by HPLC. HPLC size-exclusion chromatography revealed that oligomer preparations comprised two major peaks. High-molecular-weight AβOs are the major components in peak 1, while low-molecular-weight oligomers constitute the majority of peak 2

**Additional file 2.** Supplemental Figure 2. Characterization of AβOs in hippocampal cultures and in the frontal cortex of macaques. (A) Percentage of MAP2-positive (neuronal) and MAP2-negative (non-neuronal) cells in five independent hippocampal cultures from E17-E18 rat embryos. Total cell count was determined by DAPI staining. (B) Representative images from hippocampal neurons in culture exposed to 500 nM AβOs or vehicle for 24 h and immunolabeled for AβOs (NU4 antibody). Scale bar: 20 μm. (C) AβO binding in the frontal cortex of a macaque that received AβO injections (right panel). No AβO immunostaining was detected in the frontal cortex of the sham-operated macaques (left panel). Scale bar: 25 μm

**Additional file 3 **Supplemental Figure 3. AβOs did not cause a reduction of COX-IV expression in hippocampus of *Il1r1*^*−/−*^ mice. COX-IV messenger ribonucleic acid (mRNA) levels in the hippocampi of *Il1r1*^*−/−*^ mice 12 days after i.c.v. injection of AβOs (100 pmol). Data are expressed as means ± SEM (*n* = 6–7 animals per experimental group). Two-way ANOVA followed by Holm-Šidak post hoc test (*p* = 0.83)

## Data Availability

The datasets collected and analyzed during the current study are available from the corresponding author on reasonable request.

## Abbreviations

Actb: β-Actin
AD: Alzheimer’s disease
AβO: Amyloid-β oligomer
BBB: Blood-brain barrier
CFC: Contextual fear conditioning
Ct: Cycle threshold
DAPI: 4′,6-Diamidino-2-phenylindole
Drp1: Dynamin-related protein 1
Fis-1: Fission protein 1
IL-1: Interleukin-1
IL-1β: Interleukin-1 beta
IL-1R: Interleukin-1 receptor
*Il1r1*^*−/−*^: IL-1 receptor knockout mice
i.c.v.: Intracerebroventricular
KO: Knockout
LPS: Lipopolysaccharide
Mdivi-1: Mitochondrial Division inhibitor 1
MFN-1: Mitofusin 1
MFN-2: Mitofusin 2
mRNA: Messenger ribonucleic acid
NLRP3: Nod-like receptor family, pyrin domain containing 3
NOR: Novel object recognition task
OPA-1: Optic atrophy 1
PSD-95: Postsynaptic density protein 5
PBS: Phosphate-buffered saline
ROS: Reactive oxygen species
RNA: Ribonucleic acid
RT-qPCR: Reverse transcription quantitative real-time polymerase chain reaction
WT: Wild-type
