# Supplementary figures and images for: Interleukin-1β mediates alterations in mitochondrial fusion/fission proteins and memory impairment induced by amyloid-β oligomers
Source: J Neuroinflammation. 2021 Feb 21;18:54. doi: 10.1186/s12974-021-02099-x (PMC7897381; doi:10.1186/s12974-021-02099-x)

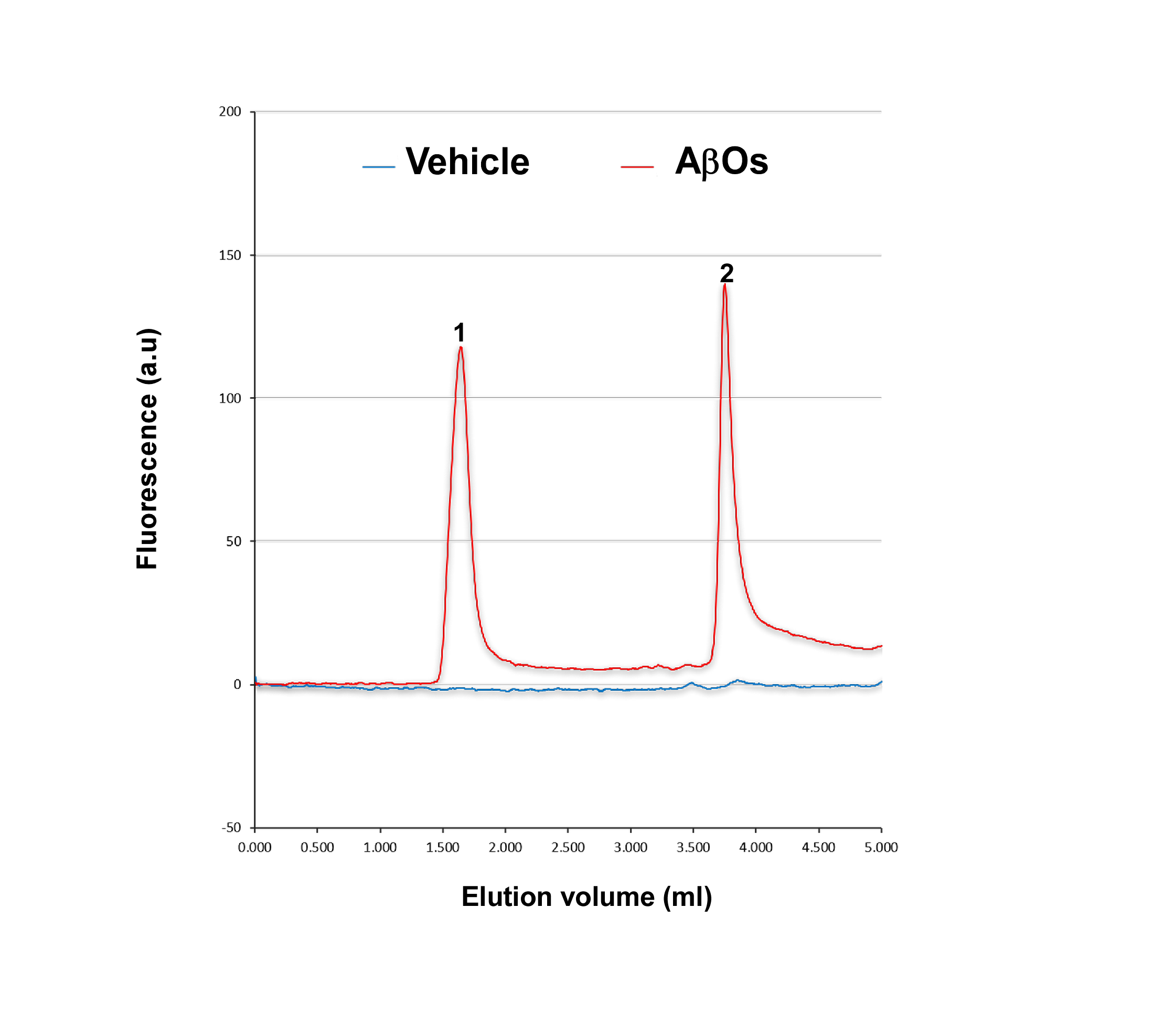

Supplement: Supplementary file 1 — Additional file 1. Supplemental Figure 1. Characterization of AβOs by HPLC. HPLC size-exclusion chromatography revealed that oligomer preparations comprised two major peaks. High-molecular-weight AβOs are the major components in peak 1, while low-molecular-weight oligomers constitute the majority of peak 2 [file 12974_2021_2099_MOESM1_ESM.tif]

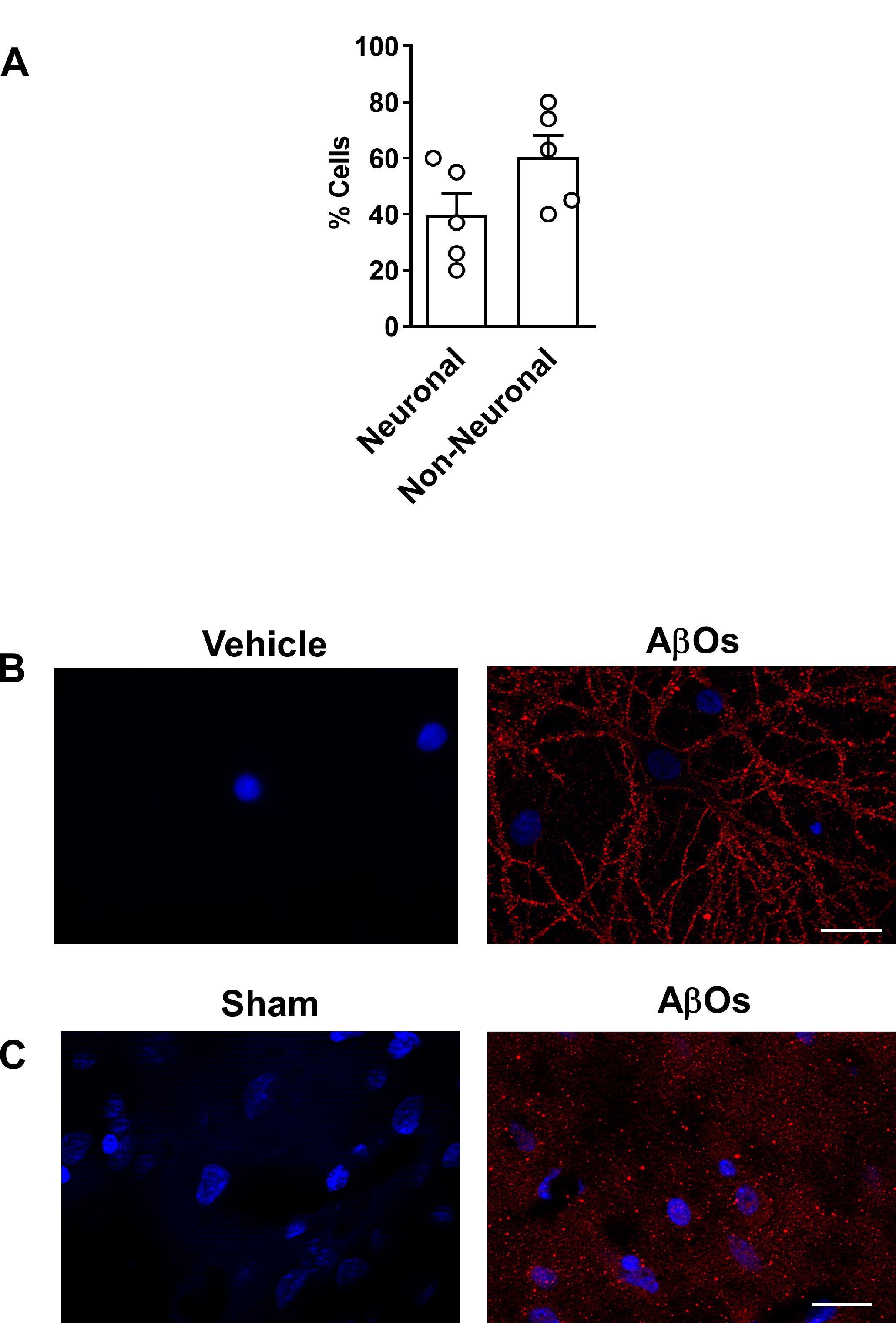

Supplement: Supplementary file 2 — Additional file 2. Supplemental Figure 2. Characterization of AβOs in hippocampal cultures and in the frontal cortex of macaques. (A) Percentage of MAP2-positive (neuronal) and MAP2-negative (non-neuronal) cells in five independent hippocampal cultures from E17-E18 rat embryos. Total cell count was determined by DAPI staining. (B) Representative images from hippocampal neurons in culture exposed to 500 nM AβOs or vehicle for 24 h and immunolabeled for AβOs (NU4 antibody). Scale bar: 20 μm. (C) AβO binding in the frontal cortex of a macaque that received AβO injections (right panel). No AβO immunostaining was detected in the frontal cortex of the sham-operated macaques (left panel). Scale bar: 25 μm [file 12974_2021_2099_MOESM2_ESM.tif]

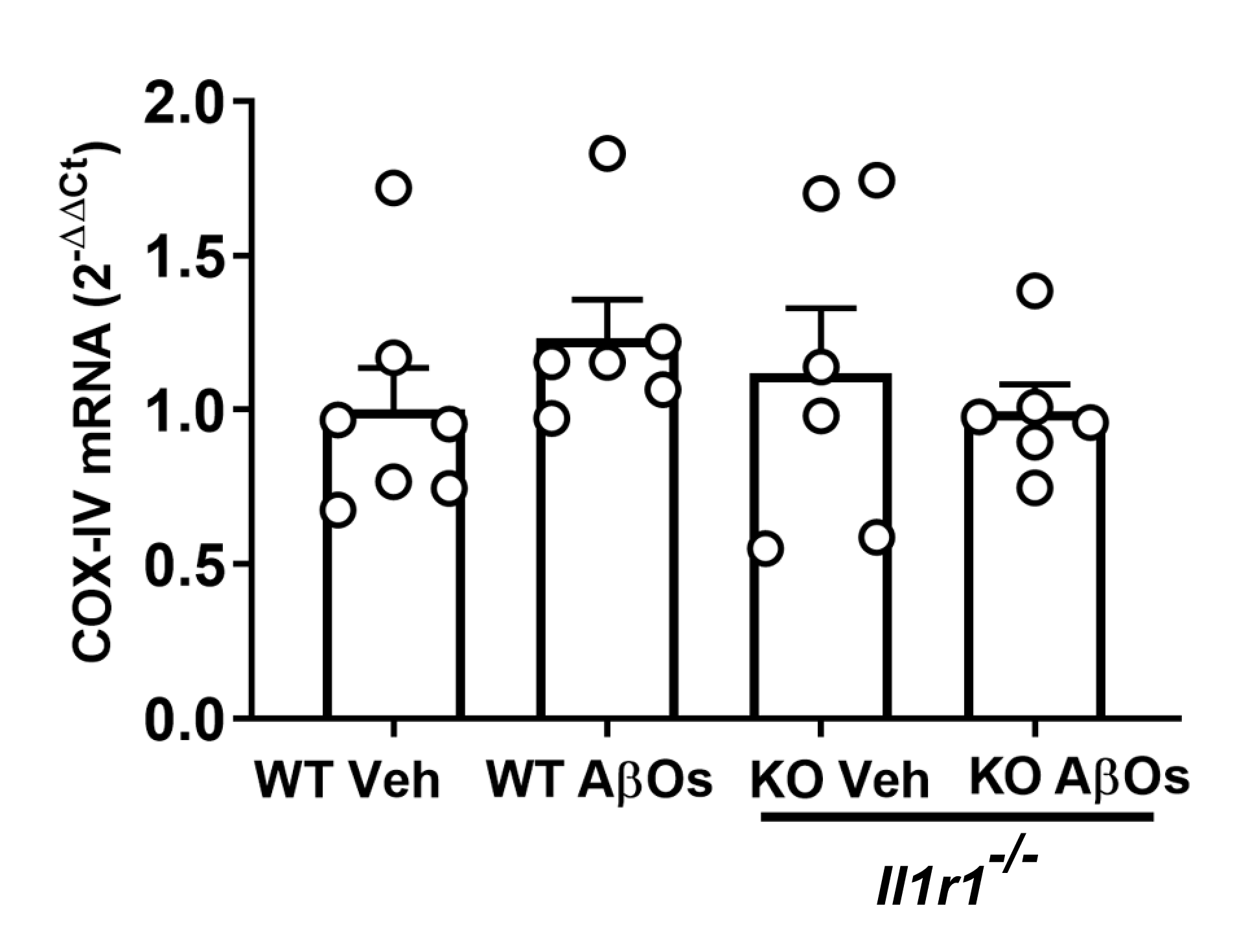

Supplement: Supplementary file 3 — Additional file 3 Supplemental Figure 3. AβOs did not cause a reduction of COX-IV expression in hippocampus of Il1r1−/− mice. COX-IV messenger ribonucleic acid (mRNA) levels in the hippocampi of Il1r1−/− mice 12 days after i.c.v. injection of AβOs (100 pmol). Data are expressed as means ± SEM (n = 6–7 animals per experimental group). Two-way ANOVA followed by Holm-Šidak post hoc test (p = 0.83) [file 12974_2021_2099_MOESM3_ESM.tif]
